# Whole-genome characterization of Candidozyma auris isolates from a Korean tertiary care centre: clade II predominance, partly unexplained fluconazole resistance and a clade I isolate

**DOI:** 10.1099/mgen.0.001802

**Published:** 2026-11-03

**Authors:** Jae Hyeon Park, Jee-Soo Lee, Seung Won Chae, Taek Soo Kim, Moon-Woo Seong

**Affiliations:** 1 Department of Laboratory Medicine, Seoul National University Hospital, Seoul, Republic of Korea; 2 Department of Laboratory Medicine, Seoul National University College of Medicine, Seoul, Republic of Korea; 3 Cancer Research Institute, Seoul National University College of Medicine, Seoul, Republic of Korea

**Keywords:** antifungal resistance, *Candidozyma auris*, whole-genome sequencing

## Abstract

In South Korea, *Candidozyma auris* is dominated by ear-associated clade II isolates, but institution-based genomic data remain limited. We analysed 35 archived isolates from 29 patients at a tertiary care centre between 2017 and 2023 using antifungal susceptibility testing and whole-genome sequencing. Thirty-four isolates belonged to clade II, and one urine isolate recovered after inter-hospital transfer belonged to subclade Ib. Serial clade II isolates from the same patient clustered together and had lower pairwise SNP distances than different-patient pairs (median, 8.5 versus 181). Among the first isolates from 29 patients, 14 (48.3%) were fluconazole resistant. In first clade II isolates, recurrent curated coding variation was concentrated in *TAC1B* and *ERG11*; *TAC1B* F214S and W198C and *ERG11* K143R occurred only in resistant isolates, but recognized coding variants did not account for all resistant isolates. Clade II isolates shared a broadly conserved subtelomeric deletion backbone, with variable chromosome 6 loss of selected glycosylphosphatidylinositol-anchor genes. The clade Ib isolate carried *ERG11* Y132F, *TAC1B* A583S and a *CDR1* V704L/E709D-like change and met CDC tentative breakpoints for resistance to fluconazole, amphotericin B and caspofungin. These findings refine the genomic profile of Korean clade II isolates and highlight the potential introduction of a distinct clade I lineage through healthcare transfer.

**Impact Statement**

*Candidozyma auris* in South Korea shows a distinctive epidemiology dominated by East Asian clade II isolates from ear specimens, but single-centre whole-genome datasets with detailed clinical context remain limited. We analysed 35 archived isolates from a Korean tertiary care centre and showed that the collection was overwhelmingly composed of clade II isolates with a broadly conserved subtelomeric deletion backbone. Serial isolates from the same patient were more closely related than isolates from different patients. Known resistance-associated coding variants explained only a subset of fluconazole-resistant clade II isolates, supporting the possibility that additional regulatory or non-coding mechanisms contribute to azole resistance in this lineage. We also identified a clade I isolate recovered after inter-hospital transfer, highlighting that genomically distinct non-clade II lineages can enter Korean tertiary care settings even where clade II predominates. These findings are relevant to genomic surveillance, resistance interpretation and infection control.

## Data Summary

Raw Illumina sequencing reads generated in this study are available in the NCBI BioProject database under accession number PRJNA1443748. Isolate-specific BioSample and SRA run accession numbers are listed in Table S1. Accession numbers for publicly available genome assemblies and SRA datasets used for phylogenetic contextualization, clade I subclade assignment, clade II-specific comparison and other comparative analyses are listed in Table S2. All software used in this study is publicly available and cited in the Methods. *De novo* genome assemblies generated in this study are publicly available in GenBank under BioProject PRJNA1443748; isolate-specific GenBank WGS accession numbers are listed in Table S1.

## Introduction

*Candidozyma auris* (formerly *Candida auris*) is an emerging healthcare-associated yeast characterized by multidrug resistance, persistence in healthcare environments and efficient nosocomial transmission [1, 2]. Whole-genome sequencing (WGS) has shown that this species comprises multiple deeply diverged clades with distinct geographic distributions and clade-associated resistance patterns. Among them, the East Asian clade (clade II) has been associated predominantly with ear-related isolates and shows characteristic chromosomal rearrangements and subtelomeric deletions enriched for adhesin and other cell-surface protein genes, features that may contribute to phenotypic differences from outbreak-associated non-clade II lineages [3, 4].

In Korea, *C. auris* has shown a distinctive epidemiology dominated by ear-associated isolates. Kwon *et al*. analysed 61 isolates from 13 hospitals and found that all belonged to clade II, only four were bloodstream isolates, fluconazole resistance was common and *ERG11* mutations were infrequent [5]. Jung *et al*. similarly reported that all 79 patients with *C. auris* at a large tertiary centre had positive ear discharge cultures, underscoring the close association between Korean *C. auris*, which has been predominantly clade II, and otologic disease [6]. More recently, Byun *et al*. [7] analysed 104 isolates from 13 Korean hospitals and showed that clade II remained predominant, but clade I isolates had also emerged, including the first reported nosocomial clade I cluster in Korea [7]. In that study, clade II isolates exhibited reduced virulence traits compared with non-clade II isolates, and *TAC1B* alterations were implicated as a major fluconazole resistance mechanism. In parallel, an intrahospital outbreak originating from a transferred patient underscored the potential for rapid hospital spread of *C. auris* in Korean high-risk settings [8].

Recent Korean genomic reports have expanded the available WGS literature but also sharpened the remaining questions. Kim *et al*. [9] reported two clade II skin isolates from a patient with atopic dermatitis [9], while Lee *et al*. [10] performed comparative phylogenomics on 39 Korean clade II isolates and suggested that high-level fluconazole resistance may, in some cases, reflect non-coding or regulatory mechanisms rather than known coding mutations alone [10]. In addition, otology-focused clinical data suggest that ear-associated *C. auris* should not be regarded as uniformly trivial, because its isolation in surgical patients has been associated with worse postoperative outcomes [11].

Despite these advances, longitudinal single-centre genomic epidemiology data from Korea remain limited. In particular, the within-centre phylogenetic structure of archived isolates, within-patient genetic relatedness, local subtelomeric variation among clade II strains and the proportion of fluconazole-resistant isolates explained by known resistance-associated variants have not been fully described. We therefore performed WGS of 35 *C*. *auris* isolates recovered from 29 patients at a tertiary care centre in South Korea. Our aims were to define their phylogenetic distribution and genetic relatedness, examine resistance-associated genomic variation and subtelomeric deletions and describe the clinical context of a clade I isolate detected after inter-hospital transfer.

## Methods

### Study design and isolate collection

This retrospective laboratory-based study included 35 archived *C. auris* isolates recovered from 29 patients at a tertiary care centre in Seoul, South Korea, between May 2017 and January 2023. Of the 35 isolates, 34 were recovered from ear discharge and one from urine. Four patients had multiple isolates; two patients had two isolates each and two patients had three isolates each. Cryopreserved isolates were subcultured on Sabouraud dextrose agar at 30 °C before phenotypic and genomic analyses. For patient-level antifungal susceptibility analyses, only the first isolate from each patient (*n*=29) was included to avoid overrepresentation of serial isolates.

### Species identification

Species identification was performed by MALDI-TOF MS using a microflex LT instrument (Bruker Daltonics, Bremen, Germany) and the MALDI Compass Library (DB9607, v10.0) on MALDI Biotyper Compass software v4.1. Identification was accepted at the species level for score values ≥2.0 according to the manufacturer’s instructions. For comparison with routine laboratory identification, results obtained using VITEK-2 YST ID cards (bioMérieux, Marcy-l’Étoile, France) with software v9.02 were also reviewed.

### Antifungal susceptibility tests

Antifungal susceptibility testing (AFST) was performed for all 35 isolates using the Sensititre YeastOne YO10 panel (TREK Diagnostic Systems, Independence, OH, USA). Minimum inhibitory concentrations (MICs) were read after 24 h of incubation at 35 °C. For patient-level antifungal susceptibility summaries, only the first isolate from each patient (*n*=29) was included to avoid overrepresentation of serial isolates. MICs for fluconazole, amphotericin B and echinocandins were interpreted using the US Centers for Disease Control and Prevention (CDC) tentative breakpoints for *C. auris* [12]. MICs for other antifungal agents were reported descriptively without categorical interpretation.

### DNA extraction, library preparation and sequencing

Genomic DNA was extracted from colonies grown on Sabouraud dextrose agar using the MagNA Pure 96 system (Roche Diagnostics, Mannheim, Germany). Genomic DNA libraries were prepared using the Nextera XT DNA Library Preparation Kit (Illumina, San Diego, CA, USA) according to the manufacturer’s protocol and sequenced on the NovaSeq 6000Dx platform to generate 2×150 bp paired-end reads. The number of raw read pairs, estimated sequencing depth, assembly statistics and accession numbers are summarized in Table S1 (available in the online Supplementary Material).

### Genome assembly and quality assessment

Raw reads were assessed using FastQC v0.12.1 and processed using fastp v1.0.1 with paired-end adapter detection and poly-G trimming enabled; reads shorter than 50 bp after trimming were discarded [13, 14]. *De novo* assemblies were generated from trimmed reads using SPAdes v4.2.0 with the --isolate option [15]. For *de novo* assembly only, trimmed reads were normalized to a target k-mer depth of 100 using BBNorm from BBTools prior to assembly [16]. Assembly quality was assessed using QUAST v5.3.0 and Benchmarking Universal Single-Copy Orthologs (BUSCO) v6.0.0 with the Saccharomycetes odb12 dataset [17, 18]. Following NCBI submission quality-control review, non-target contaminant contigs were removed, and mitochondrial contigs were excluded from the deposited assemblies. Assembly statistics in Table S1 were calculated from the final deposited assemblies.

### Phylogenetic contextualization and clade/subclade assignment

For phylogenetic contextualization, *de novo* assemblies from study isolates were analysed together with publicly available representative *C. auris* genomes from the major clades (Table S2). Core-genome alignment was performed using Parsnp v2.1.5 with B8441v3 as the reference genome. A maximum-likelihood phylogeny was inferred using IQ-TREE 3 v3.1.0 with ModelFinder and 1,000 ultrafast bootstrap replicates [19]. The resulting tree was visualized and annotated using iTOL v7 [20]. Clade assignment of study isolates was determined from their phylogenetic placement relative to the representative clade genomes.

To further resolve the phylogenetic placement of the single clade I study isolate, SLM_CA34 was analysed separately with 13 publicly available clade I isolates previously assigned to subclades Ia (*n*=2), Ib (*n*=5) and Ic (*n*=6) [4] (Table S2). Raw Illumina reads available for the comparator isolates were processed and assembled using the same quality control, read processing and *de novo* assembly pipeline applied to the study isolates, while published reference assemblies were used for the designated reference strains. Core-genome alignment and maximum-likelihood phylogenetic inference were performed using the same Parsnp and IQ-TREE workflow described above, with B8441v3 as the reference genome. The previously reported subclade assignments of the comparator isolates were used to assess the placement of SLM_CA34.

To examine within-clade II relationships at higher resolution, the 34 clade II study assemblies were subsequently analysed together with the B11220 reference genome and the Korean comparator strain B11809. Core-genome alignment and maximum-likelihood phylogenetic inference were performed using the same Parsnp and IQ-TREE settings described above, with B11220 as the reference genome. The resulting tree was annotated with collection year, patient/serial-isolate status, fluconazole phenotype among first isolates and recurrent *ERG11* and *TAC1B* coding variants.

### Clade-specific SNP analysis

After clade assignment, read-based SNP analyses were performed separately within each clade using clade-matched reference genomes. Trimmed reads from clade I isolates were analysed relative to the B8441v3 reference genome, whereas trimmed reads from clade II isolates were analysed relative to the B11220 reference genome. Variants were identified using Snippy v4.6.0 [21]. Core SNP alignments and pairwise SNP distance matrices were generated using snippy-core and snp-dists, respectively. Pairwise SNP distances were summarized separately for same-patient and different-patient isolate pairs.

### Resistance-associated variant and subtelomeric deletion analysis

To minimize reference-dependent calling artefacts, reads from all 35 isolates, including the single clade I isolate and 34 clade II isolates, were analysed independently against both the clade I reference B8441v3 and the clade II reference B11220 using Snippy v4.6.0. The targeted analysis was restricted to genes with established relevance to antifungal resistance, including fluconazole-associated genes *TAC1B*, *ERG11*, *CDR1*, *MRR1*, *MDR1* and *UPC2*, the echinocandin resistance gene *FKS1*, flucytosine-associated genes *FCY1*, *FCY2*, *FUR1* and *ADE17* and amphotericin B-associated genes *ERG3* and *ERG6*.

For each target gene, reference-specific coding coordinates, strand orientation and coding sequences were manually curated in both B8441v3 and B11220. Variants within each curated coding interval were extracted directly from sample-level snps.vcf files rather than relying solely on GFF-based annotation. Called nucleotide variants were applied to the corresponding reference coding sequence to reconstruct isolate-specific coding sequences, which were subsequently translated to determine protein-level consequences. Variant and amino acid representations obtained from the two reference-based analyses were then compared. Discordant calls attributable to differences in reference sequence, gene coordinates, local insertions or deletions or protein annotation were manually reviewed at the nucleotide and translated amino acid levels. Following this review, discordant calls were reconciled to a single curated protein-level representation or excluded from the final variant set when they could not be resolved confidently. When the affected amino acid region was supported but an exact protein-level representation could not be reconciled across references, the change was reported using a site-level ‘-like’ designation.

For clade II isolates, subtelomeric deletions were assessed separately by mapping trimmed reads to the B8441v3 reference genome, followed by copy-number analysis using CNVnator v0.3.3; candidate deletions were visually confirmed in IGV v2.19.7 [22, 23]. To complement this structural analysis, the presence of 19 selected subtelomeric adhesin/glycosylphosphatidylinositol (GPI)-anchor genes, including B9J08_004110 and B9J08_004112, was screened using MyDbFinder 2.0, a blast-based tool for querying user-defined databases. A custom database was constructed from reference sequences obtained from the Candida Genome Database and published reports of clade II subtelomeric deletions [24]. *De novo* assembled genomes were analysed using thresholds of 95% sequence identity and 60% coverage, and genes meeting both criteria were scored as detected.

### Statistical analysis

Categorical variables were summarized as frequencies and percentages and continuous variables as medians with interquartile ranges (IQRs). Statistical analyses were performed using R v4.5.2.

### Ethics statement

This study was approved by the Institutional Review Board of Seoul National University Hospital (IRB No. H-2301-063-1393). The requirement for informed consent was waived because archived isolates and retrospective clinical data were analysed.

## Results

### Isolates, species identification and genome overview

A total of 35 archived *C. auris* isolates recovered from 29 patients were available for WGS. Of these, 34 isolates (97.1%) were recovered from ear discharge and 1 (2.9%) from urine. All isolates were identified as *C. auris* by MALDI-TOF MS, whereas all were misidentified as *Candida duobushaemulonii* by the VITEK-2 system. *De novo* assemblies were of sufficient quality for downstream analyses, with a median of 454 contigs (IQR, 421–498), a median N50 of 73,330 bp (IQR, 65,460–77,694) and a median complete BUSCO score of 95.9% (IQR, 95.7–96.2%; minimum, 94.5%).

### Phylogenetic structure and within-patient relatedness

Phylogenetic analysis including publicly available reference genomes assigned 34 study isolates to clade II and 1 urine isolate (SLM_CA34) to clade I (Fig. 1). In a separate clade I-specific phylogeny, SLM_CA34 clustered with the selected clade Ib comparator isolates, supporting its assignment to subclade Ib (Fig. S1). In the clade II-specific phylogeny, serial isolates recovered from the same patient formed closely related clusters (Fig. 2). Consistent with this pattern, median pairwise SNP distances were lower for same-patient isolate pairs than for different-patient isolate pairs, with median distances of 8.5 SNPs (IQR, 6.5–12.0; range, 4–19) across eight same-patient comparisons and 181 SNPs (IQR, 169–197; range, 7–243) across 553 different-patient comparisons (Fig. S2). For contextual comparison, in a separate clade II SNP matrix including the Korean strain B11809, the study isolates showed median pairwise distances of 201.5 (IQR, 195.0–218.8; range, 185–254) to B11220 and 154.5 (IQR, 139.5–172.5; range, 87–206) to B11809; all 34 study isolates were closer to B11809 than to B11220.

**Fig. 1.**
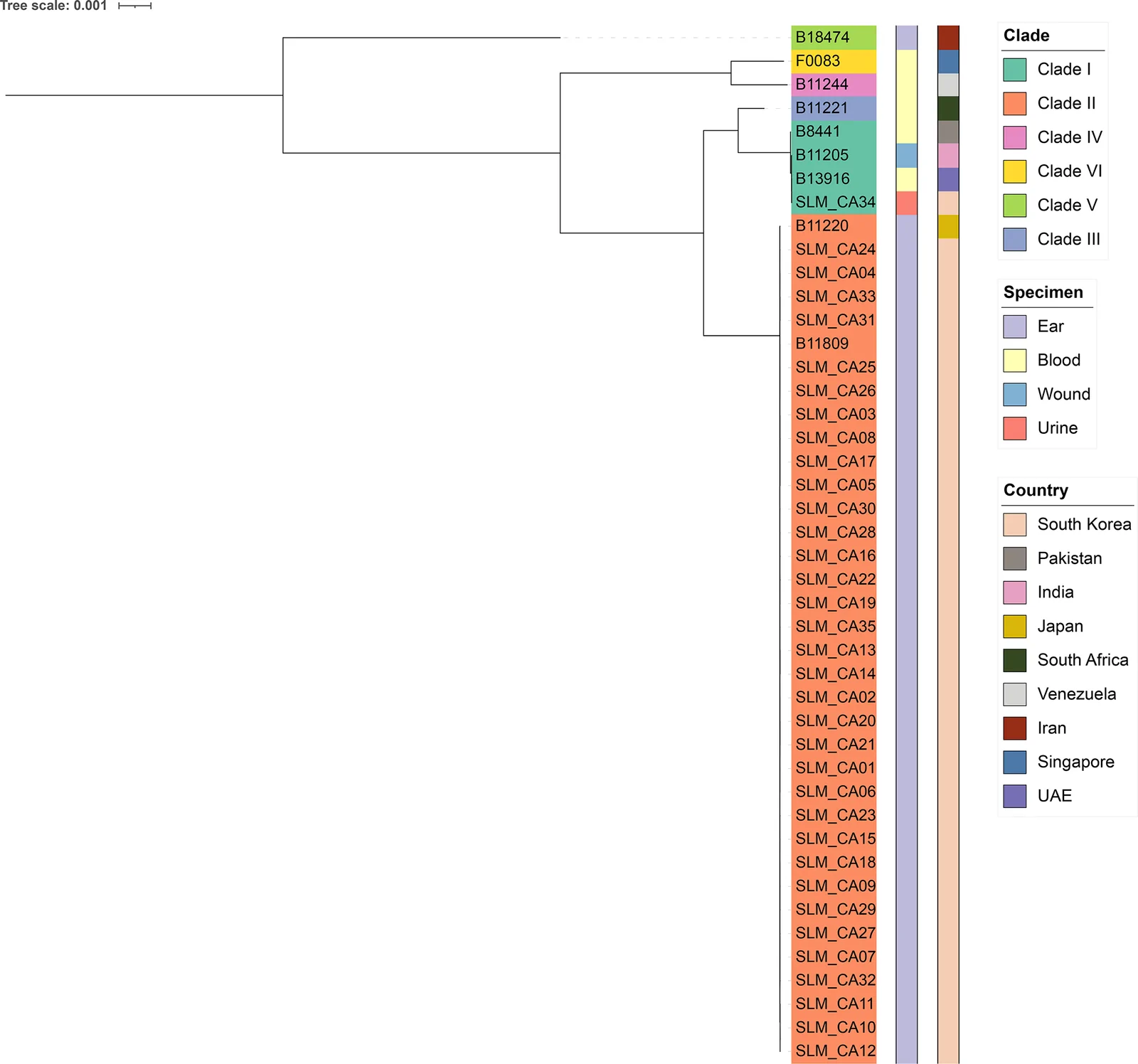
Phylogenetic contextualization of *C. auris* study isolates. A maximum-likelihood phylogeny was inferred from a core-genome alignment of 35 study isolates and nine publicly available representative genomes from the major clades. Branch lengths are proportional to the number of nucleotide substitutions per site, and the scale bar represents 0.001 substitutions per site. Adjacent annotation tracks indicate clade, specimen type and country of origin.

**Fig. 2.**
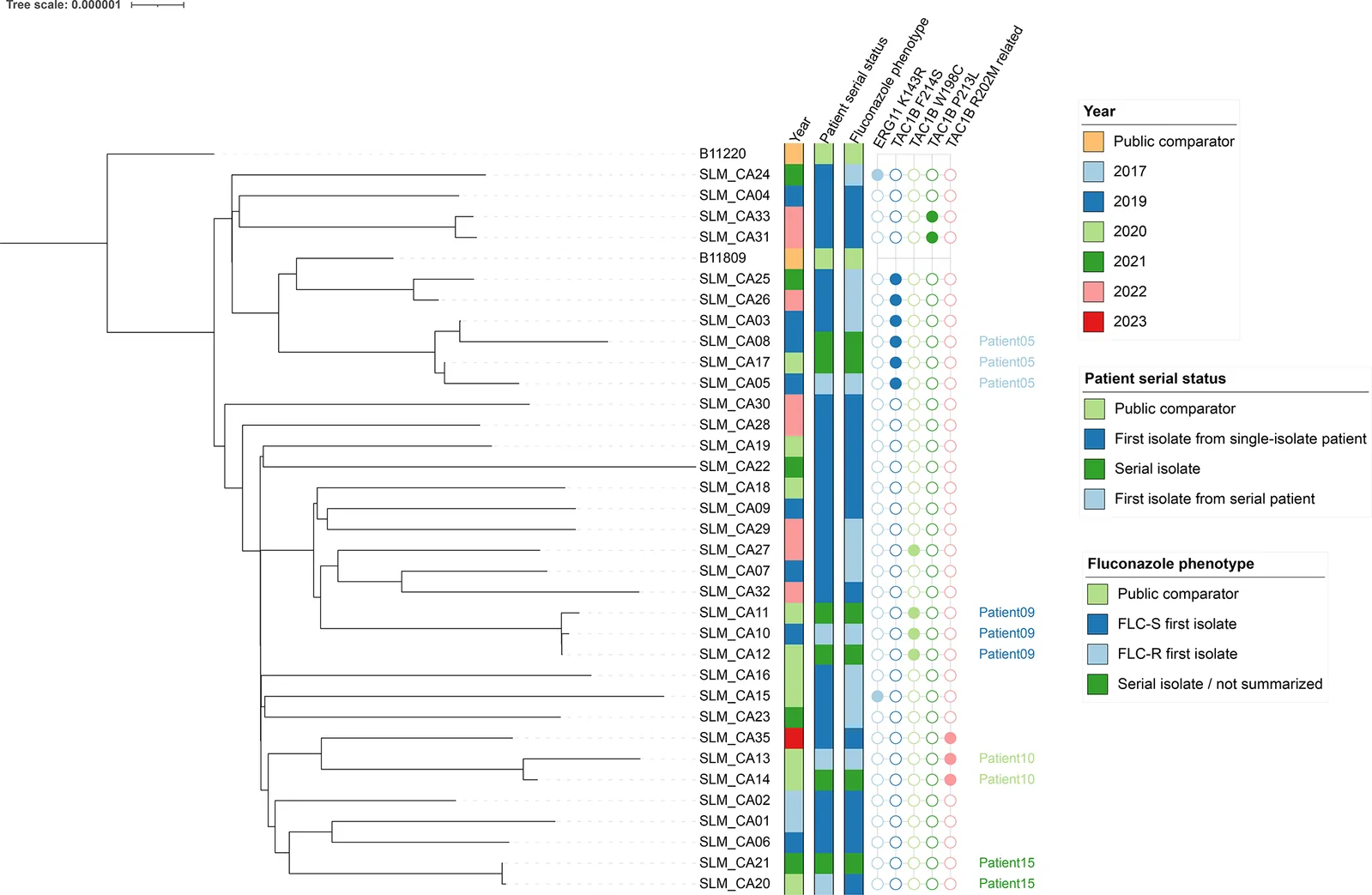
Clade II-specific phylogeny and associated metadata. The maximum-likelihood tree was inferred from a Parsnp core-genome alignment of 34 clade II study isolates, the B11220 reference genome and the Korean comparator strain B11809. Branch lengths are proportional to nucleotide substitutions per site, and the scale bar represents 1×10^−6^ substitutions per site. Annotation tracks indicate collection year, patient sampling status, fluconazole phenotype among first isolates and recurrent *ERG11* and *TAC1B* variants. Filled and open circles indicate variant presence and absence, respectively. Matching label colours identify serial isolates from the same patient. FLC-R, fluconazole resistant; FLC-S, fluconazole susceptible.

### Antifungal susceptibility and resistance-associated variation

AFST results were available for all 35 isolates. To avoid overrepresentation of serial isolates, patient-level summary analyses were restricted to the first isolate from each patient (*n*=29; Table 1), whereas individual isolate-level MIC values for all isolates are provided in Table S4. Among the first isolates, the fluconazole MIC distribution had an MIC50 of 16 mg l^−1^ and an MIC90 of 256 mg l^−1^, and 14/29 isolates (48.3%) met the CDC tentative breakpoint for fluconazole resistance. Amphotericin B resistance and caspofungin resistance were each observed in one first isolate (1/29, 3.4%). Across all 35 isolates, none met the CDC tentative resistance breakpoints for anidulafungin or micafungin (Table S4). The single clade I urine isolate was categorized as resistant to fluconazole, amphotericin B and caspofungin according to CDC tentative interpretive criteria.

**Table 1.** Antifungal susceptibility profiles of the first *C. auris* isolate recovered from each patient (*n*=29)

Antifungal agent	MIC50 (mg l^−1^)	MIC90 (mg l^−1^)	MIC range (mg l^−1^)	%S	%R
Fluconazole	16	256	4 to >256	51.7	48.3
Voriconazole	0.12	2	0.015 to >8	–	–
Posaconazole	0.06	0.5	0.015 to >8	–	–
Itraconazole	0.12	0.5	0.06 to >16	–	–
Amphotericin B	0.5	1	0.25 to 2	96.6	3.4
Caspofungin	0.12	0.25	<0.008 to >8	96.6	3.4
Anidulafungin	0.12	0.25	<0.015 to 0.25	100	0
Micafungin	0.12	0.12	0.03 to 0.25	100	0
Flucytosine	0.5	1	0.12 to >64	–	–

MIC values are expressed in mg l−1. MIC50 and MIC90 denote the MICs inhibiting 50% and 90% of isolates, respectively. Susceptibility categories are shown for fluconazole, amphotericin B and echinocandins according to CDC tentative breakpoints for *C. auris*; MICs for other agents are presented descriptively. Individual isolate-level MIC values are provided in Table S4.

R, resistant; S, susceptible.

In patient-level analysis restricted to the first clade II isolate from each patient (*n*=28), curated coding variants in fluconazole-associated genes were stratified by fluconazole phenotype (Table 2). Because variants were not mutually exclusive, isolate-level co-occurrence patterns are provided in Table S5. After manual curation of dual-reference Snippy calls, *TAC1B* F214S (4/28) and W198C (2/28) were observed only among fluconazole-resistant first isolates, whereas *TAC1B* P213L (2/28) was observed only among fluconazole-susceptible first isolates. *TAC1B* R202M-related alleles were observed in both susceptible and resistant backgrounds. *ERG11* K143R was identified in two fluconazole-resistant first isolates. Fluconazole-resistant first isolates and recurrent *ERG11* and *TAC1B* variants were distributed across multiple branches of the clade II phylogeny rather than forming a single cluster (Fig. 2). Other curated coding variants in fluconazole-associated genes were infrequent. No canonical *FKS1* hotspot mutation was detected, and no retained resistance-associated coding variant was identified in the targeted flucytosine- or amphotericin B-associated genes.

**Table 2.** Curated coding variants in fluconazole-associated genes among first clade II isolates, stratified by fluconazole phenotype (*n*=28)

Gene	Curated variant	Total, n/28	FLC-S, n/15	FLC-R, n/13	Evidence status
*ERG11*	K143R	2/28	0/15	2/13	Previously reported fluconazole resistance-associated substitution
*TAC1B*	F214S	4/28	0/15	4/13	Previously reported fluconazole resistance-associated substitution
*TAC1B*	W198C	2/28	0/15	2/13	*TAC1B* is associated with fluconazole resistance; variant-level evidence is not established
*TAC1B*	P213L	2/28	2/15	0/13	No established variant-level resistance association
*TAC1B*	R202M-related allele	2/28	1/15	1/13	No established variant-level resistance association
*TAC1B*	Other FLC-R-only singleton alleles (S192N, I196_W198del, L582I, I645L, S427R)	5/28	0/15	5/13	No established variant-level resistance association; each variant was observed once
*TAC1B*	Other alleles observed only in FLC-S isolates (D462N/M653I, L625F/G867V, complex allele)	3/28	3/15	0/13	No established variant-level resistance association
*CDR1*	Y469*	1/28	1/15	0/13	No established variant-level resistance association
*CDR1*	V114-region substitution	1/28	0/15	1/13	No established variant-level resistance association
*CDR1*	K1185-region substitution	1/28	1/15	0/13	No established variant-level resistance association
*MDR1*	M377T	1/28	1/15	0/13	No established variant-level resistance association

Counts are patient level and are based on the first clade II isolate from each patient. Variants were not mutually exclusive, and counts across variant rows are therefore not additive. Only genes with at least one retained curated coding variant are shown; no curated coding variant was detected in *MRR1* or *UPC2*. Isolate-level variant co-occurrence patterns are provided in Table S5.

FLC-R, fluconazole resistant; FLC-S, fluconazole susceptible.

The clade I isolate (SLM_CA34) was confirmed to carry *ERG11* Y132F, *TAC1B* A583S and a *CDR1* V704L/E709D-like change, whereas no other curated resistance-associated coding variants were identified in the targeted panel.

### Subtelomeric deletions among clade II isolates

Subtelomeric deletion sizes across the 34 clade II isolates remained tightly clustered at most chromosome ends after manual curation (Fig. 3), supporting a conserved clade II subtelomeric backbone. Several chromosome ends showed little or no between-isolate variation, including Chr3-beg (12 kb), Chr4-beg (8 kb), Chr5-beg (15 kb) and Chr5-end (50–51 kb). In contrast, greater heterogeneity was observed at selected ends, most notably Chr6-beg, where deletion sizes ranged from 35 to 47 kb. Most isolates (26/34, 76.5%) showed a 35 kb deletion at Chr6-beg, whereas 8/34 (23.5%) had larger deletions.

**Fig. 3.**
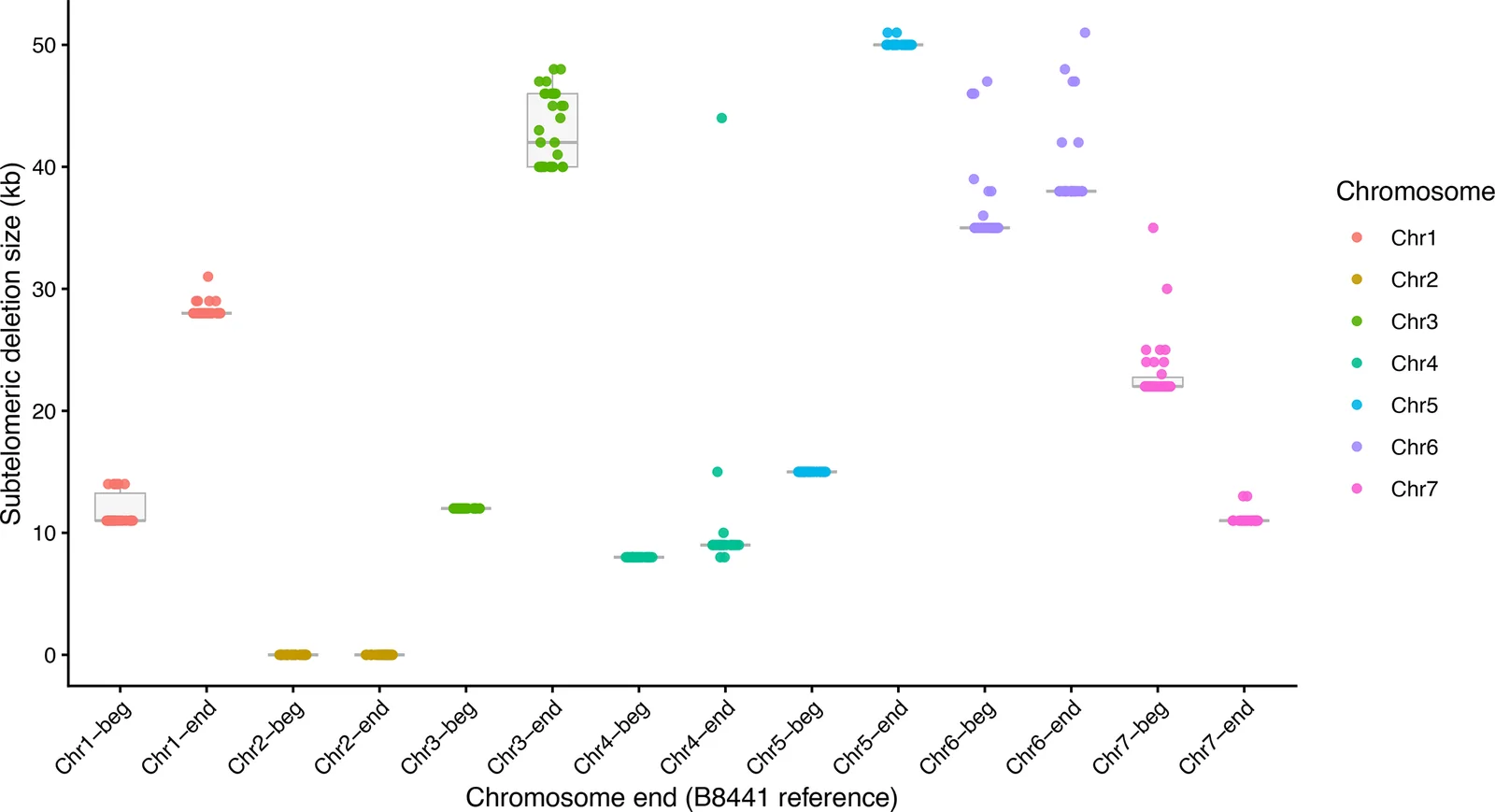
Subtelomeric deletion patterns in clade II *C. auris* isolates, with variable loss at the beginning of chromosome 6 (*n*=34). Grey boxplots summarize isolate-level subtelomeric deletion sizes at each chromosome end. The central line indicates the median, the box limits indicate the IQR, and the whiskers extend to the most extreme values within 1.5× the IQR. Coloured overlaid points represent individual isolates. X-axis labels indicate the beginning (beg) or end (end) of each chromosome in the B8441 reference assembly used for copy number variation analysis. Most chromosome ends showed similar deletion sizes across isolates, whereas greater heterogeneity was observed at Chr6-beg.

In a complementary MyDbFinder screen using 95% sequence identity and 60% coverage, most selected subtelomeric adhesin/GPI-anchor genes showed conserved clade II detection patterns (Table S3A and S3B). Within-clade variability was concentrated at the chromosome 6 subtelomere: B9J08_004110 was detected in 26/34 (76.5%) isolates, whereas B9J08_004112 (ALS5) was detected in 30/34 (88.2%) isolates. Non-detection of one or both genes corresponded to isolates with more extensive chromosome six subtelomeric deletion on CNVnator/IGV analysis.

### Clinical context of the clade I isolate

The clade I isolate was recovered from urine in a 3-year-old boy who had been transferred from another hospital after prolonged ventilator care with tracheostomy. A day-1 surveillance culture from an endotracheal aspirate was later identified as *C. auris*, but that isolate was not archived and was therefore unavailable for genomic analysis. *C. auris* was subsequently isolated from urine on hospital day 28 and on two additional occasions. No further *C. auris* isolates from non-otologic sites were identified in the hospital, including the patient’s ward, during the following year.

## Discussion

In this longitudinal single-centre WGS study, the archived *C. auris* isolates analysed at our institution were overwhelmingly composed of clade II isolates recovered from ear specimens, whereas one isolate belonged to clade Ib. Serial isolates recovered from the same patient were more closely related to each other than to isolates from different patients, supporting within-patient relatedness within this collection. Together, these findings provide institution-based genomic data on Korean clade II isolates and a clinically contextualized example of a non-clade II isolate.

This study was designed to characterize the genomic features of clade II isolates in a single institutional collection and to analyse a non-clade II isolate in clinical context. Earlier Korean studies had already established that *C. auris* in Korea was strongly associated with ear specimens and was predominantly assigned to clade II, while later reports showed that non-clade II isolates, including clade I, had also emerged and could be associated with nosocomial spread [5–8]. More recent WGS studies provide a clearer framework for interpreting institutional datasets such as ours [9, 10]. Within that context, our study adds genome-level data on Korean clade II isolates, including resistance-associated variation and subtelomeric deletion patterns, and provides a clinically contextualized example of a non-clade II isolate recovered after inter-hospital transfer.

The predominance of ear isolates in this study is informative primarily from a genomic perspective. East Asian clade II isolates are known to be associated with ear-related specimens and to show characteristic chromosomal rearrangements and subtelomeric deletions compared with non-clade II lineages [3, 4, 25]. These structural features have been proposed to contribute to phenotypic differences between clade II and outbreak-associated non-clade II lineages [4]. Korean data further suggest that clade II isolates may show reduced virulence traits relative to non-clade II isolates, but they should not be regarded as uniformly benign [7]. In addition, recent otology-focused data suggest that *C. auris* detected in surgical patients may be associated with worse postoperative outcomes [11]. Our subtelomeric findings are consistent with this clade II genomic backbone while also suggesting limited within-clade heterogeneity, particularly at a chromosome 6-associated subtelomeric locus. Notably, this signal remained evident under the more stringent MyDbFinder thresholds used in this study, with isolates showing more extensive structural loss also tending to lack detection of selected subtelomeric adhesin/GPI-anchor genes [4, 25]. The within-patient relatedness observed in our serial isolates is biologically plausible, but the absolute SNP distances should be interpreted cautiously. Recent outbreak investigations have shown much smaller SNP ranges among epidemiologically linked isolates in some settings, but no universal threshold can be applied across all *C. auris* datasets [26]. This is particularly relevant because SNP distances in *C. auris* are influenced by clade background, sampling interval and the bioinformatic workflow used for variant calling and filtering [27]. Accordingly, the pairwise SNP distances reported here are better interpreted as internally consistent relative measures within our dataset than as hard thresholds for transmission inference.

One of the main biological messages of this study is that fluconazole resistance in Korean clade II remains only partly explained by currently recognized coding variants. In patient-level analysis restricted to the first clade II isolate from each patient, recurrent curated coding variation was dominated by *TAC1B*, whereas *ERG11* substitutions were infrequent, broadly consistent with earlier Korean findings [5, 7]. This pattern is also concordant with functional work supporting *TAC1B* as a major determinant of clinical fluconazole resistance in *C. auris* [28]. However, curated coding variants accounted for only a subset of fluconazole-resistant clade II isolates in our collection, indicating that currently recognized coding determinants did not fully explain the observed resistance phenotype. This supports the emerging view that azole resistance in *C. auris* may additionally involve broader regulatory or non-coding mechanisms affecting efflux and ergosterol-related pathways beyond the canonical coding variants typically screened in routine genomic analyses [10, 29]. In this context, variants such as *TAC1B* W198C, *TAC1B* I196_W198del and *TAC1B* L582I should currently be regarded as candidate rather than established resistance determinants. The distribution of fluconazole-resistant first isolates and recurrent *ERG11* and *TAC1B* variants across multiple clade II branches suggests that these phenotypes and variants were not confined to a single phylogenetic sublineage. However, formal inference of homoplasy would require denser sampling and ancestral-state reconstruction and was beyond the scope of this study.

Our subtelomeric results also refine the interpretation of structural variation within clade II. Most clade II isolates in our collection shared the broad subtelomeric deletion pattern expected for the East Asian lineage [4, 25]. However, a subset showed more extensive loss near the beginning of chromosome 6, corresponding to the absence of selected GPI-anchor protein genes. This suggests that Korean clade II isolates preserve the broader clade-defining structural framework while retaining isolate-level genomic diversity that may still be biologically relevant [4]. Whether these differences influence persistence, adaptation to the ear or clinical behaviour remains uncertain and would require additional functional study. In a contextual clade II SNP matrix including the Korean strain B11809, all study isolates were closer to B11809 than to B11220. This observation should nevertheless be interpreted cautiously, because B11809 was included as an assembly-based external comparator and inclusion of external genomes changed the size of the shared core alignment. Accordingly, our data do not support firm inference regarding the direction of dissemination or the role of any single hospital as the source of broader spread, which would require denser national sampling, harmonized reference-based SNP analyses and stronger epidemiological linkage data [10, 25].

Our clade I case is best interpreted in the context of importation risk rather than chronological priority. In the clade I-specific phylogeny, SLM_CA34 clustered with the selected subclade Ib comparator isolates, providing finer phylogenetic resolution than major-clade assignment alone. However, this placement does not establish the geographic origin or a direct transmission source, given the selected comparator set and the absence of genomes from the referring hospital. Non-clade II *C. auris* has already been documented in Korea, including clade I isolates and a nosocomial clade I cluster [7]. A recent multicentre Korean MLST and sequence-based survey further showed the coexistence of endemic clade II isolates with emerging clade I isolates across Korean hospitals, including clade I candidemia clusters during 2022–2024 [30]. Korean outbreak data have also shown that a transferred index patient can seed intrahospital spread [8]. Internationally, multiple introductions followed by intra- and inter-hospital transmission have been documented in several healthcare systems [31–34]. Accordingly, our case supports the view that genomically distinct non-clade II lineages can enter Korean tertiary-care settings through healthcare transfer, even where archived institutional collections are dominated by clade II isolates. This finding supports heightened surveillance and prompt infection-control assessment of non-clade II isolates, particularly in patients transferred from other healthcare facilities.

The phenotype of this clade I isolate should nevertheless be described conservatively. The presence of *ERG11* Y132F and *TAC1B* A583S makes the fluconazole-resistant phenotype biologically plausible, but the absence of recognized echinocandin- or amphotericin B-resistance determinants leaves the non-azole phenotype less readily explained. This is particularly important because interpretation of caspofungin MICs in *C. auris* may be confounded by paradoxical growth or the Eagle effect [35, 36]. It is therefore safer to describe this strain as resistant to fluconazole, amphotericin B and caspofungin according to current tentative criteria rather than as definitively pan-resistant unless repeat or orthogonal susceptibility testing confirms broader echinocandin resistance. This conservative interpretation is also supported by Korean nationwide data, in which clade I isolates remained susceptible to amphotericin B and the echinocandins [7].

The universal misidentification of our isolates by VITEK-2 also remains practically relevant. Although MALDI-TOF MS correctly identified all isolates, the routine phenotypic platform misidentified them as *C. duobushaemulonii*. This finding reinforces the importance of accurate species-level identification for surveillance, timely infection-control measures and appropriate interpretation of unusual resistance profiles, especially in settings where clade II isolates are common and non-clade II introductions may be overlooked [5, 37].

This study has several limitations. It was performed at a single centre and was based on an archived isolate collection dominated by ear isolates, most of which were recovered in the outpatient setting. Accordingly, the collection should not be interpreted as a direct representation of current inpatient transmission dynamics or the overall institutional burden of *C. auris*. The non-clade II finding was based on a single archived isolate. Our genomic analyses relied on short-read data and focused primarily on coding variation, so promoter alterations, transcriptional changes, aneuploidy or more complex structural events may have been missed. In addition, because SNP distances in *C. auris* can vary according to analysis workflows, our pairwise distance estimates should be interpreted as internally consistent relative measures rather than universal epidemiologic thresholds [27]. Finally, because genomes from the referring hospital and environmental isolates were unavailable, we could not reconstruct the origin or onward transmission potential of the clade I isolate with confidence.

Taken together, our results add institution-based genomic data to the evolving Korean literature on *C. auris*. The study provides a focused whole-genome characterization of archived Korean clade II isolates and a clinically contextualized analysis of a non-clade II isolate. Overall, its main contribution is to refine the genomic picture of Korean clade II isolates while highlighting that genomically distinct non-clade II strains can also be encountered in tertiary-care practice.

## Supplementary material

10.1099/mgen.0.001802 Supplementary Material 1.

10.1099/mgen.0.001802 Supplementary Material 2.

## Abbreviations

AFST: antifungal susceptibility testing
BUSCO: Benchmarking Universal Single-Copy Orthologs
CDC: Centers for Disease Control and Prevention
GPI: glycosylphosphatidylinositol
IQR: interquartile range
MALDI-TOF MS: matrix-assisted laser desorption/ionization time-of-flight mass spectrometry
MIC: minimum inhibitory concentration
SNP: single-nucleotide polymorphism
WGS: whole-genome sequencing
