# Supplementary material for: Whole-genome characterization of Candidozyma auris isolates from a Korean tertiary care centre: clade II predominance, partly unexplained fluconazole resistance and a clade I isolate
Source: Microb Genom. 2026 Nov 3;12(11):001802. doi: 10.1099/mgen.0.001802 (PMC13730534; doi:10.1099/mgen.0.001802)
Supplement: Supplementary file 1 [file mgen-12-01802-s001.pdf]

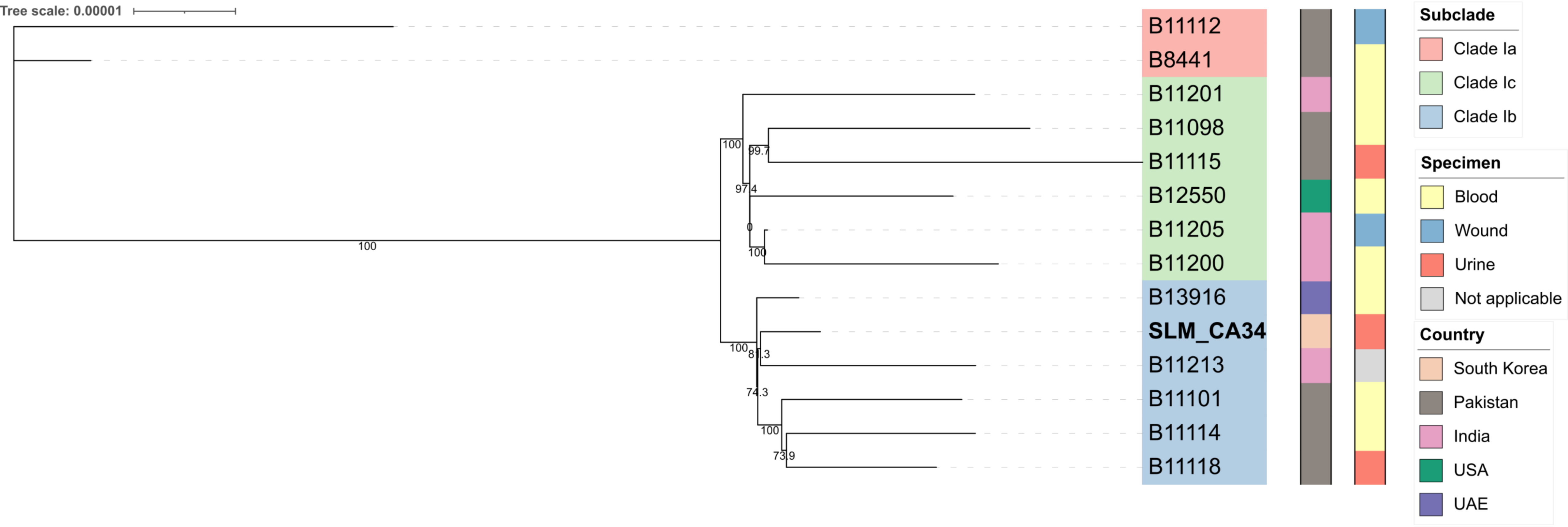

Fig. S1. Clade I-specific phylogenetic placement of the study isolate SLM_CA34.
A maximum-likelihood phylogeny was inferred from a Parsnp core-genome alignment of SLM_CA34 and 13 publicly available *Candidozyma auris* clade I comparator isolates previously assigned to subclades Ia (n=2), Ib (n=5), and Ic (n=6). B8441v3 was used as the reference genome for core-genome alignment, and phylogenetic inference was performed using IQ-TREE 3 with ModelFinder and 1,000 ultrafast bootstrap replicates. Branch lengths are proportional to the number of nucleotide substitutions per site, and the scale bar represents 1 × 10−5 substitutions per site. Numbers at internal nodes indicate ultrafast bootstrap support values (%). Colored backgrounds behind strain labels indicate previously reported subclade assignments for comparator isolates and the inferred placement of SLM_CA34. Adjacent annotation tracks indicate specimen type and country of isolation. The study isolate SLM_CA34 is shown in bold and clustered with the selected subclade Ib comparator isolates, supporting its assignment to subclade Ib.

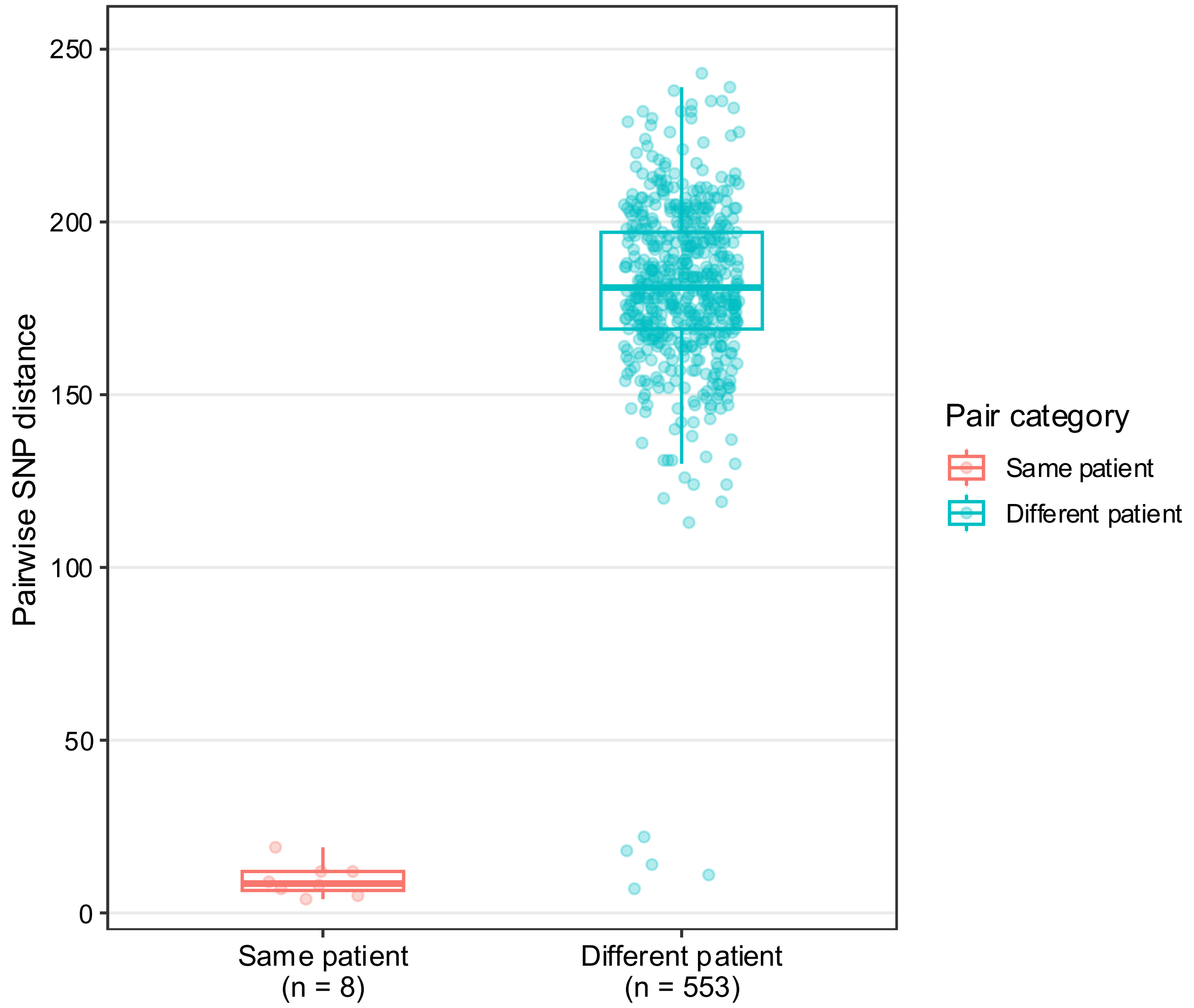

Fig. S2. Pairwise SNP distances among clade II *Candidozyma auris* isolates for same-patient and different-patient isolate pairs.
Pairwise SNP distances were calculated from the study-only clade II SNP matrix generated using B11220 as the reference genome after excluding external comparator genomes. Each point represents one pairwise isolate comparison. The central line indicates the median, box limits indicate the interquartile range, and whiskers extend to the most extreme values within 1.5× the interquartile range; all pairwise observations, including values beyond the whiskers, are shown as points.
